# Chimeric recombinant rotavirus-like particles as a vehicle for the display of heterologous epitopes

**DOI:** 10.1186/1743-422X-6-192

**Published:** 2009-11-06

**Authors:** Andrea Peralta, Paula Molinari, Oscar Taboga

**Affiliations:** 1 Consejo Nacional de Investigaciones Científicas y Técnicas (CONICET). Av. Rivadavia 1917 (c.p.1033) Ciudad Autónoma de Buenos Aires, Argentina; 2 Instituto de Biotecnología, Instituto Nacional de Tecnología Agropecuaria (INTA), cc25 (c.p.1712) Castelar, Buenos Aires, Argentina

## Abstract

In order to improve the presentation and immunogenicity of single epitopes, virus-like particles (VLPs) are being used as platforms for the display of foreing epitopes on their surface. The rotavirus major capsid protein VP6 has the ability to self-assemble into empty non-infectious VLPs. In the present study, we analyzed the use of double layered VLPs (made up of VP2 and VP6 rotavirus proteins) as carriers to display a 14 amino acid epitope fused to three different aminoacidic regions of VP6 exposed on the surface of VLPs. Although all chimeric protein were correctly expressed in insect cells, only one of them resulted in spontaneous assembly of VLPs displaying the heterologous epitope on their surface, confirmed by sandwich ELISA and electron microscopy. Furthermore, the injection of chimeric VLPs into mice elicited higher antibody titers than the monomeric chimeric protein. Our results identify an specific amino acid region of VP6 which allows the insertion of at least a 14 amino acid heterolgous epitope and demonstrate its potential as immunogenic carrier.

## Background

Virus-like particles (VLPs) are complexes composed of viral structural proteins that retain the ability to self-assemble without requiring the presence of the viral genome, mimicking the overall structure of virus particles. They are considered as safe and non-infectious tools for several purposes such as diagnostic assays [1-4], cell interaction studies [5,6] and vaccines [7-14]. VLPs are commonly more immunogenic than subunit or recombinant immunogens based on single, monomeric proteins, and are able to stimulate both the humoral and cellular pathways of the immune system. VLPs offer a promising approach to the production of vaccines against many diseases, because their repetitive, high density display of epitopes is often effective in eliciting strong immune responses [15]. This is further enhanced by the particulate nature of VLPs, especially in the size range of around 40 nm that appears to be optimal for uptake of nanoparticles by dendritic cells [16].

VLPs provide the spatial structure for the display of conformational epitopes and can be exploited as platforms for the presentation of foreign epitopes or targeting molecules on chimeric VLPs. This can be achieved via transcriptional fusion of heterologous sequences and viral proteins in such a way that the chimeric protein is assembled into VLP during *de novo *synthesis.

Rotaviruses, members of the *Reoviridae *family of segmented, dsRNA genome, are the most important cause of viral gastroenteritis in infants and young animals around the world [17-19]. Members of this family are nonenveloped, with complex capsids containing several concentric protein layers displaying icosahedral symmetry. Rotaviruses have a triple concentric capsid. The innermost layer, which is composed of VP2 protein, encloses the different genomic segments of dsRNA together with VP1 and VP3 proteins. The middle layer is composed of 780 molecules of VP6 protein, which are distributed as 260 trimers. The outermost layer is composed of glycoprotein VP7 and spikes of dimers of VP4 [17]. Both outer proteins are the targets for neutralizing antibodies and define the virus G and P serotypes, respectively. VP6 is the most abundant protein in the virus particle, comprising about of 51% of the total protein mass [17].

Co-expression of VP2 and VP6 in both mammalian and insect cells results in the production of VLPs that can be easily purified [20,21], whereas the expression of VP2 alone results in the production of pseudo-core particles or CLPs [22,23]. Although the formation of VLPs requires the presence of VP2, VP6 alone can form spherical or tubular aggergates [24] and could be overexpressed and purified in large quantities. VP6 self-assembles into different types of particles depending on conditions such as pH, ionic strength and divalent cation concentration.

Although VP6 is part of the middle layer of the rotavirus mature particles, it elicits a strong humoral immune responses after rotavirus infection. At least one strong Th cell epitope has been mapped, which is highly conserved in most group A rotavirus strains studied so far and it was proposed that Th cells specific for VP6 epitopes may constitute an important proportion of the total polyclonal Th cell response against a porcine rotavirus in spleen cells [25].

Based on the study of VP6 priming immune responses to the external rotavirus proteins, Esquivel et al suggested that the VP6-specific Th cells can provide cognate help to B cells specific for neutralizing epitopes on the VP7 and/or VP4 molecules, and that this help could be heterotypic [26]. On the other hand, synthetic peptides spanning different regions of VP6 protein were able to elicit high titers of antibodies [27,28].

Together, the ability of VP6 to form multimeric structures and the strong immune responses that VP6 can elicit in different species point at VP6 in an excellent candidate as a carrier for foreign epitopes. In this report, we used the baculovirus expression system to produce recombinant VLPs based on rotavirus capsid VP6 and VP2 proteins from SA11 strain of simian rotavirus. We first searched for positions in the surface loops of VP6 that could accommodate a foreign 14 amino-acid peptide derived from the simian paramyxovirus 5 (V5 epitope, [29]) without affecting capsid formation and then we examined whether the heterologous sequences inserted into these sites were displayed on the surfaces of the recombinant VLPs.

## Results

### Election of possible insertion sites into VP6 sequence

**Figure 1:**
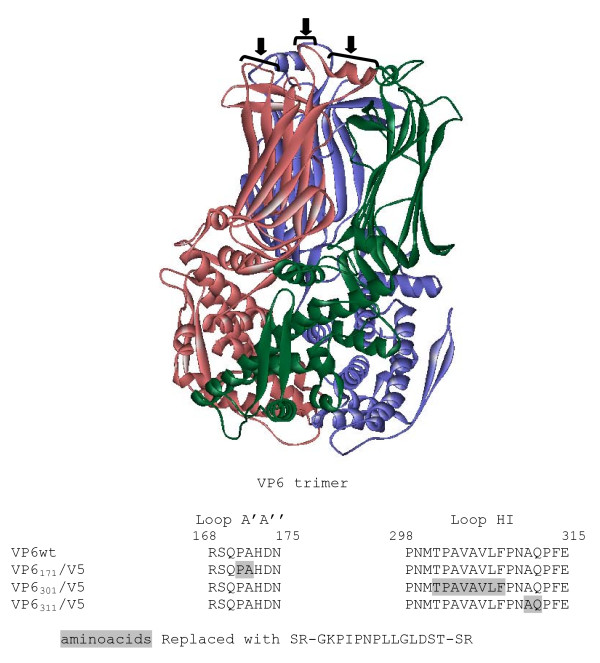
**Insertion of the epitope V5 into VP6 sequence**. A solid ribbon drawing the VP6 trimer is shown at the top. Arrows and brackets show only in one chain the amino acids regions of VP6 protein selected in order to insert a heterologous epitope. In the lower panel, partial amino acid sequences of VP6 and a series of VP6 mutants were created by replacing the amino acids residues indicated by grey boxes with the V5 epitope flanked by SR residues.

SA11 VP6 protein is 97% identity to RF VP6, In turn, RF VP6 structural conformation is well known (PDB code: 1qhd). In order to select possible insertion sites into VP6 sequence, we analyzed the spatial conformation of 1qhd and three regions located in loops exposed onto VLPs surface were selected: the amino acids 171-172 into loop A'A" and amino acids 311-312 and 301 to 308 both in loop HI. Selected regions are shown in Figure 1.

### Effect of epitope insertion on recombinant expression and antigenic properties of monomers

In order to evaluate the effect of epitope insertion on the expression, interaction and antigenicity of the recombinant VP6 proteins, insertion mutants were constructed bearing 14 amino-acids of the V5 epitope at different positions and expressed in the baculovirus system.

Extracts of Sf9 cells infected with the different recombinant baculoviruses exhibited an intense band in Coomassie-stained polyacrylamide gels, at the electrophoretic mobility expected for the recombinant proteins and absent in extracts of mock-infected Sf9 cells (data not shown). The apparent molecular weights of the VP6 variants were slightly higher than wild-typeVP6 and reached similar expression levels.

**Figure 2:**
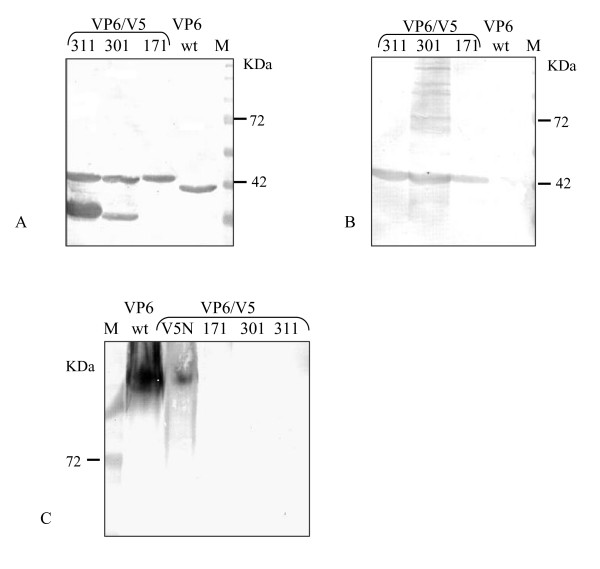
**Expression of chimeric VP6/V5 protein**. Sf9 cells infected with baculovirus AcVP6, AcVP6_171_/V5, AcVP6_301_/V5 or AcVP6_311_/V5, were analyzed by western blot using a polyclonal sera anti-VP6 (A) or a MAb anti-V5 epitope (B). In order to determine the trimerization ability of the chimeric VP6/V5, cells extracts were analyzed by electrophoresis under native conditions and western blot (C).

To confirm the identity of the chimeric proteins, a monoclonal antibody to V5 epitope and an anti-VP6 rabbit polyclonal serum were employed in Western blot experiments. The extracts of Sf9 cells infected with all the recombinant baculoviruses exhibited a band with the expected electrophoretic mobility that strongly reacted with the monoclonal antibody and was absent in the extracts of Sf9 AcVP6-infected (Figure 2A), suggesting that the antigenicity of the V5 epitope was manteined in all the mutants. The same intense bands were observed when the polyclonal rabbit serum directed to rotavirus was used (Figure 2B), but it was possible to observe a partial proteolysis in mutants VP6_301_/V5 and VP6_311_/V5 which was only partially prevented with the addition of aprotonine and leupeptinA.

As VP6_311_/V5 showed a pronounced cleavage, we continued this work only with VP6_171_/V5 and VP6_301_/V5.

### Effect of epitope insertion on trimer and multimer formation

In order to evaluate the implicances of the insertion of V5 sequence on the ability of VP6 mutants to oligomerize into trimers, total extracts from Sf9 cells infected with the different recombinant baculoviruses were disrupted at 37°C instead of 100°C, resolved by gel electrophoresis (in no-reducing or native conditions) and monomers and trimers detected by Western blot using a SA11 polyclonal rabbit serum or a V5 monoclonal antibody. As control of trimers, VP6wt and mutant VP6-V5N were used. This mutant bears the epitope V5 in the amino terminal of VP6 as it has been reported that insertions at N-terminal of VP6 did not affect the ability to oligomerize into trimers (Reddy et al 1992). As previously reported [30], wild type VP6 was only detected as trimers in these conditions (Figure 2C). In the same way, mutant VP6-V5N was detected as trimers. However, we could not detect trimers from insertional mutants VP6_171_/V5 or VP6_301_/V5.

The ability of the insertional mutants to form multimeric structures was first evaluated by their ability to sediment through 30% sucrose cushions. Lysates of Sf9 infected cells from the different recombinant baculoviruses AcVP6/V5 were ultracentrifugated and the pellets were disrupted and resolved by SDS-PAGE and Western blot. The results showed that mutants VP6_171_/V5 and VP6_301_/V5 were able to interact in some multimeric form.

### Effects of epitope insertion on VLP assembly and exposition of the heterologous sequence

**Figure 3:**
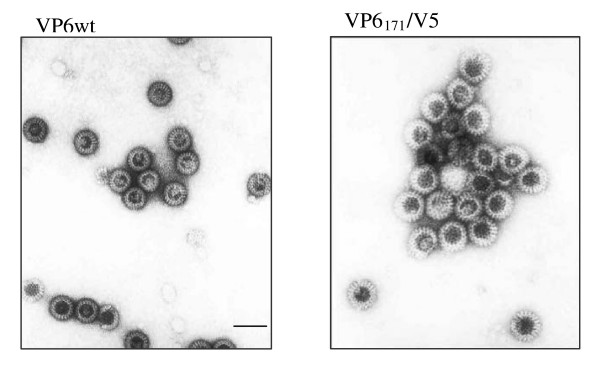
**Negative staining of wt or recombinant VLPs**. In order to characterize the VLPs formation, supernants of Sf9 cells co-infected with recombinant baculoviruses expressing VP2, VP6wt or VP6_171_/V5 or VP6_301_/V5 were processed as described in Materials and Methods. Particles were adsorbed onto carbon-coated grids, staining with 2% uranyl acetate and examined immediately. Bar indicates 100 nm.

In order to determine the ability of the different VP6 mutants to interact with VP2 to form VLPs, supernatans of co-infections were analyzed by CsCl gradients and electron microcopy. By electron microscopy observation, only VP6_171_/V5 was able to form VLPs. These VP6_171_/V5 VLPs were indistinguishable from wild type VLPs, and the amount of VLPs per field was very similar (Figure 3). VP6_171_/V5 VLPs also exhibited mean diameter comparable with those of VLPs VP6 (Table 1).

**Table 1:** Mean diameter of VLPs

**VLPs (6 + 2)**	**MEAN DIAMETER**
VP6wt	63 ± 2,6 nm

VP6_171_/V5	69 ± 3,4 nm

These values were obtained through analysis of 100 particles by electron microscopy.

**Figure 4:**
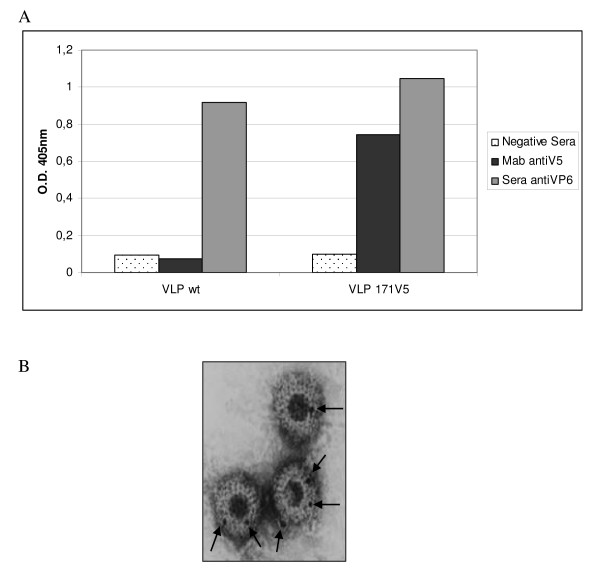
**Characterization of recombinant VLPs VP6_171_/V5**. A) Sandwich ELISA to detect epitope exposition onto recombinant VLPs surface. Purified wt and recombinant VLPs were captured by polyclonal sera against SA11 strain, then were detected by a monoclonal antibody anti-V5 peptide or a polyclonal mouse sera against VP6 protein. B) Microscopy electronic and immunogold labeled. Purified recombinant VLPs VP6_171_/V5 were loaded onto nickel grids were incubated with a MAb anti-V5 and then labeled with a rabbit anti-mouse conjugated with gold.

In order to determine the epitope exposition on VLPs VP6_171_/V5 surface, a sandwich ELISA was performed. The results suggested that V5 was exposed on the surface of these VLPs (Figure 4A)

To further confirm these results, purified VP6_171_/V5 VLPs were immunolabeled with MAb anti-V5 and revealed with an anti-mouse IgG conjugated to gold particles. Figure 4B shows that VP6_171_/V5 VLPs were specificly labeled on their surface.

### Immunogenic features of the VLPs VP6_171_/V5 mutant

As high quantities of VP6_171_/V5 were routinely obtained rendering VLPs with V5 epitope exposed to the surface, we decided to continue the experiments of immunization only with this mutant. The immunogenicity of VP6_171_/V5 VLPs was compared with the same chimeric protein as a monomer by innoculating groups of five mice with each preparation. VLPs of wild type VP6 were used to immunize control mice. Antibody titers elicited to anti V5 epitope following two immunizations are given in Table 2. High levels of anti-V5 (~3 log_10_) antibodies were measured after 20 days after booster when as little as 300 ng of chimeric VLPs were administered, representing 9 ng of V5 peptide. In contrast, it was necessary to innoculate 5 μg of chimaeric monomers to elicit detectable antibody titers to V5, suggesting that the multimeric presentation of V5 on the surface of the VLP strongly enhanced their immunogenicity.

**Table 2:** Anti-V5 epitope antibody titers elicited in mice immunized with VLPs VP6_171_/V5 or monomeric VP6_171_/V5, measured by ELISA

**Immunogen**	**Mean log_10 _ELISA endpoint titer**
VLPs	3.08 ± 0.28 (2.6-3.2) †
Monomers	1.88 ± 0.40 (1.7-2.6)

Titers were expressed as log_10 _of the reciprocal of the highest serum dilution, which give OD readings al least twofold higher than those of control animals. † Ranges of mean titers are shown in brackets.

## Discussion

We demonstrated the influence of the insertion of a 14 amino acids heterologous sequence in the ability of VP6 from a simian rotavirus strain to trimerize, multimerize and interact with VP2 to render chimaeric VLPs. In particular, when the V5 sequence was inserted at position 171 of VP6, VLPs were indistinguishable of recombinant 2/6 particles, with the inserted epitope protruding from the particle surface. In addition, these chimeric VLPs elicited higher antibody titers to the inserted epitope than the monomeric chimeric protein, suggesting that these chimerical VLPs may be a useful vaccine approach for the multimeric presentation of immunogenic epitopes.

The morphogenesis of rotavirus in mammalian cells has been widely studied (López et al., 2005), although some aspects remain less understood. Rotavirus VP6 protein plays a central rol in virus morphogenesis by establishing interactions with other structural proteins as well as to the nonstructural protein NSP4. Core particles assembled from VP1, VP2 and VP3 acquire an icosahedral layer of VP6 arranged in trimeric units. Double-shelled particles are then translocated to the lumen of the endoplasmic reticulum via NSP4 and virus assembly is completed by the incorporation of VP4 and VP7. The obtention of rotavirus-like particles by infection or co-infections of insect cells by recombinant baculovirus has provided strong evidences on the site of assembly of the major rotavirus structural proteins VP2 and VP6 and in the characterization of functional domains. VP2 forms pseudocores of 50 nm of diameter composed by 60 dimers in the cytoplasm of infected insect cells observed as aggregates [31], while VP6 alone forms tubular structures composed by a variable number of trimers [24]. However, when both recombinant proteins are simultaneously expressed, VP6 drastically changes its localization [31] and self-assembly, rendering double-layered VLPs that are assembled inside the insect cell and can be recovered from the supernatants of co-infected cultures.

By the *in vitro *transcription and translation analysis of truncated forms of VP6, a large domain involved in trimerization have been previously characterized spanning amino-acidic positions 105 to 328 [32]. By using VP6 deletion mutants produced in mammalian cells by recombinant vaccinia virus, the trimerization domain could be delimited to a region between amino-acids 246 and 314 [30], and the N terminal end of this domain was postulated between amino-acids 147 and 246. Subsequently, by using X-ray crystallography approaches [33] it was postulated that the amino acids 171 and 172 of VP6 are involved in intra-trimer contacts. In this work, we observed that the insertion of the V5 sequence in mutant VP6_171 _affects trimer formation. This result supports the hypothesis about the extension of the N-terminal end of the trimerization domain, extending it at least to the amino-acid 171.

The analysis of the three-dimensional structure of VP6 from rotavirus group A using X-ray crystallography [33] determined that the region proposed as a trimerization domain is localized within a bigger domain formed for β-strands, denominated domain H (residues 151 to 334). This amino-acidic organization is characterized by its rigidity, and any insertion affecting the β-strand structure may also affect trimer formation. However, bioinformatic analysis of VP6/V5 chimeras (Swiss prot and Modeller, results not show) has predicted that the V5 insertions at positions 171-172, 311-312 and a replacement at positions 301-308 do not drastically affect the structure of domain H of the VP6 monomer. However, it was possible to obtain recombinant VLPs VP6-V5 only when the heterolgous epitope was inserted into 171-172 residues. Possibly, the insertion of an heterologous sequence in this particular amino acidic region of VP6, unstabilizes or prevents the formation of trimers, but the presence of VP2 protein counteracts this negative effect and helps in the formation of stable VLPs. This fact agrees with the results obtained by X-ray crystallography where the correct geometry of the viral particle is determined by VP2 [24,33,34].

Rotavirus-derived VLPs were first assessed as an immunological carrier by Redmond and collegues in 1991. They demonstrated that peptides coupled to VP6 spheres elicited a greater humoral response than other traditional carriers or peptides alone, and that this carrier was not supressed by the pre-existence of anti-rotavirus antibodies [27,28]. We demonstrated the influence of the insertion of an heterologous sequence in the ability of simian rotavirus VP6 to trimerize, multimerize and interact with VP2 to render chimeric VLPs. In particular, when the V5 sequence was inserted at position 171-172 of VP6, VLPs were indistinguishable of recombinant 2/6 particles, with the inserted epitope exposed on the particle surface. In addition, these chimeric VLPs elicited higher antibody titers to the inserted epitope than the monomeric chimaeric protein, suggesting that this chimeric VLP may be useful vaccine vehicles for the multimeric presentation of immunogenic epitopes.

## Methods

### Primer design for insertional mutagenesis

A collection of mutants harboring an XbaI site at the desired VP6 (SA11 strain) positions were created by PCR using adequate primers and named according to the position of the insertion in the amino-acid sequence. The internal positions in the amino-acid sequence of VP6 SA11 strain were selected on the basis of the analyses of spatial conformation of VP6 RF strain deposited on Protein Data Bank (PDB code: 1qhd) and visualized using the WebLab Viewer software (Accelrys Software Inc.). Three regions located in loops exposed onto rotavirus capsid surface were selected. These amino acidic regions were 171-172, 311-312 and 301 to 308. The primers used for mutagenesis were: L171-for: TT**TCTAGA**TTGTGATCTATTTAGTGT, L171-rev: GG**TCTAGA**CATGATAATTTGATGGGC, L311-for: A**TCTAGA**CCATTCGAACATCAT, L311-rev: A**TCTAGA**ATTCGGGAATAGTAC, R308-for: CC**TCTAGA**CCGAATGCACAGCCATTC and R301-rev: TT**TCTAGA**CATGTTTGGTGGTCTCAG. The XbaI restriction sites are in bold.

### Construction of recombinant baculoviruses

The complete methodology for the construction of recombinant baculoviruses has been previously published as a manual [35]. The VP6 and VP2 sequences used in this work derived from the simian strain SA11. For VP2 expression, VP2 coding sequence was first subcloned into pVL1393 (Pharmingen). For VP6 mutants, PCR amplification products were obtained using pBSVP6 (bearing the entire coding sequence of VP6, kindly provided by Dr. J. Blackhall) as a template. N-terminal and C-terminal moieties were sequentially cloned into pVL1393, creating an XbaI site at the junction and replacing two residues in VP6. For all mutants, the primer VP6-for (A**AAGCTT**AACATGGATGTCCTATA, bearing a HindIII restriction site) was used as the forward primer for the N-terminal moiety and VP6 rev (T**GGTACC**TCATTTAATGAGCAT, bearing a KpnI restriction site) was used as the reverse primer for the C-terminal moiety. Once the two moieties of VP6 with XbaI sites were cloned together, a synthetic dsDNA constructed by annealing of oligonucleotides V5-For and V5-Rev (V5-for: AA**TCTAGA**GGTAAGCCTATCCCTCTCCTC, V5-rev: AA**TCTAGA**CGTAGAATCGAGACCGAGGAGAGGGTT, the XbaI restriction sites are in bold) and coding for an epitope derived from simian paramyxovirus 5 [29] was introduced in frame. An extra recombinant VP6 (called VP6-V5N) was constructed bearing the V5 epitope at the N-terminal end (The primers used were: VP6N-for: AA**CCCGGG**GATGTCCTATACTCTTTG, VP6N-rev: AA**TCTAGA**TCATTTAATGAGCATGC, V5N-for: T**CCCGGG**AGT***ATG***GCTAAGCCTATCCCTAACCCTCTCCTC, V5N-rev: T**CCCGGG**CGTAGAATCGAGACCGAGGAGAGGGTT. The XmaI restriction site sare in bold, the ATG codon is pointed in italic). Relevant sequences of the transfer plasmids were confirmed by sequencing and Sf9 insect cells were co-transfected with 2 μg of each plasmid and 0.5 μg of linear AcNPV DNA (Pharmingen) using cellfectin (Invitrogen). Resulting recombinant baculoviruses AcVP2, AcVP6, AcVP6_171_/V5 AcVP6_301_/V5 and AcVP6_311_/V5 were propagated as previously described [30]. All recombinant baculoviruses were propagated in *Spodoptera frugiperda *Sf9 cells (ATCC) grown at 27°C in TNM-FH medium (SIGMA) supplemented with 10% foetal bovine serum (FBS) and antibiotic-antimycotic solution (GIBCO).

### Expression analysis of VP6 mutants

Sf9 cells grown in 25 cm^2 ^flasks were infected at a multiplicity of infection (moi) of 5 with each recombinant baculovirus and 5 days post infection (dpi), cells were lysed by boiling in presence of 500 μl cracking buffer (50 mM Tris-HCl pH 6.8, 2% SDS, 0.01% bromo-phenol blue, 1% 2-mercaptoethanol, 10% glycerol) and proteins were resolved by SDS-PAGE. Recombinant proteins were detected by Coomassie blue staining or Western blot with a commercial monoclonal antibody (MAb) directed to the V5 epitope (Invitrogen) or a rabbit polyclonal serum against rotavirus SA11.

### Analysis of the formation of VP6 trimer, multimers and VLPs

For testing the ability of VP6 mutants to form trimers, 5 dpi infected cells grown in 25 cm^2 ^flasks were disrupted in 500 μl cracking buffer (without 2-mercaptoethanol or DTT), incubated at 37°C for 30 min and subjected to SDS PAGE under no reducing conditions [36]. Furthermore, 5 dpi infected cells were harvested in TNMC buffer, subjected to three freeze-thaw cycles, sonicated and then electrophoresed under native conditions [36]. Trimers were detected by Western blot with a MAb to V5 epitope or a rabbit polyclonal serum against rotavirus SA11.

In order to evaluate the ability of VP6 mutants to multimerize alone or interact with VP2 to form VLPs, Sf9 cells grown in four 175 cm2 flasks were infected or co-infected at a moi of 5 for each version of AcVP6 and a moi of 8 for AcVP2 and 5 dpi supernatants were collected, clarified 10 min at 1,600 × g and VLPs were pelleted by ultracentrifugation through a 30% (w/w) sucrose cushion at 70,000 × g for 90 min at 10°C. Pellets were resuspended in 300 μl of TNMC buffer (10 mM Tris-HCl pH 7, 150 mM NaCl, 1 mM MgCl_2_, 10 mM CaCl_2_). Ten μl were disrupted with cracking buffer and analyzed by SDS-PAGE and Western blot. Recombinant proteins were detected by a rabbit polyclonal serum against rotavirus SA11 or a mix of this serum and the Mab directed to V5. The remaining material was loaded onto a CsCl gradient (density - 1.32 g ml^-1^) and centrifuged 134,000 × g for 18 h at 10°C. The fraction corresponding to VLPs was collected, dialyzed against TMNC buffer and concentrated by ultracentrifugation at 70,000 × g for 90 min at 10°C. Pellets were resuspended in 50 μl of TNMC buffer and stored at 4°C for ELISA and electron microscopy observation.

### Electron microscopy

VLPs obtained by ultracentrifugation in CsCl gradients were loaded onto cupper grids of a 200 mesh, stained with 2% uranyl acetate and analyzed by electronic microscopy (JEOL SVC-model) at 80 kV.

In order to determinate the heterologous peptide exposition onto recombinant VLPs surface, an immunogold labelling was performed. All incubations were carried out on drops of reagent on strips of parafilm in a covered chamber. Briefly, recombinant VLPs obtained by ultracentrifugation in CsCl gradients were loaded onto nickel grids and a blocking step was carried out for 30 min with PBS 1% BSA. Once removed the excess buffer, the grids were incubated with a drop of monoclonal anti-V5 diluted 1/50 in blocking buffer for 1 h a room temperature. After each step, grids were washed five times with ultrapure water. Then, the samples were incubated with a drop of anti-mouse antibody conjugated to 10 nm gold particles (SIGMA) diluted 1/50 in blocking buffer. Finally, grids were stained in 2% uranyl acetate and analyzed by electron microscopy.

### Characterization of recombinant VLPs by ELISA

Microtitration plates (Maxisorp, Nunc, Rochester, NY) were coated with 50 μl of an optimal dilution (1/2000) of a rabbit polyclonal serum against rotavirus-SA11 in carbonate buffer pH 9.6 (Na_2_CO_3 _15 mM, NaHCO_3 _35 mM, pH 9,6), by an overnight incubation at 4°C. The next day, a blocking step was performed for 1 h at 37°C with PBS, 0.05% Tween-20, 5% horse serum (PBS-T-HS) and 5% non-fat milk. After each step, plates were washed five times with washing buffer (PBS, 0.05% Tween-20). Next, a 50 μl volume of a dilution of purified VP6/VP2 or recombinant VP6_171_V5/VP2 VLPs (25 ng/well) was prepared, added it to the plate and incubated for 1 h at 37°C. After this incubation, optimal sera dilutions of MAb against V5 epitope or mouse polyclonal against rotavirus SA11 were transferred to the ELISA plate and incubated for 1 h at 37°C. Finally, 50 **μ**l of a 1:2000 dilution of horseradish peroxidase conjugated antibody (KPL, Guildford UK GU2 5GN) in PBS-T-HS were applied for 1 h at 37°C. Then, 50 μl of the substrate solution (ABTS 0.5 mg/ml (Sigma-Aldrich, St. Louis, MO) in 0.1 M citrate buffer, pH 4.2 containing 0.03% hydrogen peroxide) were added, and after incubation at room temperature for 20 minutes absorbance readings at 405 nm were determined. Each sample was tested in duplicates.

### Immunization and evaluation of the humoral immune response

Female BALB/c mice (6-8 weeks old) were used for vaccination. Animals received two doses of an oil-based vaccine consisting of 300 ng of VLPsVP6_171_V5 or 5 μg of monomeric VP6_171_V5, formulated with incomplete Freund adjuvant (IFA) at days 1 and 21 by the intra-peritoneal (ip) route. A control group was immunized with VP6wt. Animals were bled at different times post-vaccination and were maintained all the time with free access to sterile food and water.

The humoral immune response against V5 epitope was evaluated by ELISA. Briefly, a recombinant not related protein CAT-V5, was produced in E. coli (using pRSET expression vector) and purified by Ni-NTA agarose (QIAGEN). Microtitration plates (Maxisorp, Nunc) were coated with 75 ng/well of purified CAT-V5 in carbonate buffer pH 9.6, by an overnight incubation at 4°C. The next day, plates were blocked with PBS-T-HS and 5% non-fat milk and subsequently incubated for 1 h at 37°C with mice serum sample (serial dilutions from 1:50 to 1:5000) and a secondary anti-mouse-IgG horseradish peroxidase conjugated antibody (KPL). Primary and secondary antibodies were diluted with PBS-T-HS. After each step, plates were washed five times with washing buffer. Finally, the reaction was developed by addition of ABTS 0.5 mg/ml (Sigma-Aldrich, St. Louis, MO) in 0.1 M citrate buffer pH 4.2 containing 0.03% hydrogen peroxide, and absorbance readings at 405 nm were determined after incubation at room temperature. Each sample was tested in duplicate.

Titers were expressed as log_10 _of the reciprocal of the highest serum dilution, which give OD readings al least twofold higher than those of control animals.

## Abbreviations

VLPs: virus-like particles; PBS-T-HS: PBS, 0.05% Tween-20, 5% horse serum; CAT-V5: Chloranphenicol Acetyl Transferase protein fused to V5 epitope.; MAb: monoclonal antibody; OD: optical density

## Competing interests

The authors declare that they have no competing interests.

## Authors' contributions

AP: constructed all recombinant baculoviruses, characterized the different chimeric VP6-V5, developed a sandwich ELISA to characterize recombinant VLPs, immunized mice and evaluated the immune response by ELISA (expressed and purified a not related recombinant protein CAT-V5 in order to evaluate the antibody title against V5 epitope). PM: established optimal conditions to obtain VLPs, prepared all samples to electron microscopy (negativ stain and immunogold labelling). OT: have been involved in drafting the manuscript and have given final approval of the version to be published.

## Author's information

A.P.: PhD. in Biology, University of Buenos Aires, Argentina. Assistant Research of Argentine Commission for Scientific and Technological Research (CONICET).

P.M.: Doctoral fellow of CONICET.

O.T.: PhD. in Biology, University of Buenos Aires, Argentina. Research of CONICET and Research of National Institute of Agricultural Technology (INTA).
